# The effect of a raw vs dry diet on serum biochemical, hematologic, blood iron, B_12_, and folate levels in Staffordshire Bull Terriers

**DOI:** 10.1111/vcp.12852

**Published:** 2020-04-23

**Authors:** Johanna Anturaniemi, Sara Zaldívar‐López, Robin Moore, Mikko Kosola, Satu Sankari, Stella M. Barrouin‐Melo, Anna Hielm‐Björkman

**Affiliations:** ^1^ Faculty of Veterinary Medicine Department of Equine and Small Animal Medicine University of Helsinki Helsinki Finland; ^2^ Faculty of Veterinary Medicine, Genomics and Animal Breeding Group Department of Genetics University of Córdoba Cordova Spain; ^3^ School of Veterinary Medicine and Zootechny Department of Anatomy, Pathology and Clinics Federal University of Bahia Salvador Bahia Brazil

**Keywords:** blood, canine nutrition, carbohydrates, fats, food processing

## Abstract

**Background:**

To date, very few studies have compared the effects of different types of feeding practices on canine physiology, such as feeding exclusively dry, raw, or homemade foods.

**Objectives:**

We aimed to report the changes in hematologic, serum biochemical, plasma folate, B_12_, and whole blood iron levels in dogs fed two different diets.

**Methods:**

A pilot study was developed to compare the effects of a heat‐processed high carbohydrate (HPHC) and nonprocessed high‐fat (NPHF) diet. A total of 33 client‐owned Staffordshire Bull Terriers were used; 18 had canine atopic dermatitis, seven were healthy, and eight were grouped as “borderline” dogs since they did not fulfill at least six of Favrot's criteria. The comparisons were made between the diet groups at the end visit of the diet intervention, as well as within the diet groups during the study.

**Results:**

Significant differences between and within the diet groups were observed, although the majority of outcomes remained within the RIs. The median time of diet intervention was 140 days. Red blood cell counts, mean cell hemoglobin concentrations, and platelet counts were significantly higher, and mean cell hemoglobin, mean cell volume, alkaline phosphatase, inorganic phosphorus, and cholesterol were significantly lower in the dogs fed the NPHF diet compared with those fed the HPHC diet after the diet trial was completed. In addition, folate, B_12_, and iron decreased significantly in the NPHF diet group.

**Conclusions:**

This pilot study indicated that diet had an impact on blood values, although most remained within RIs, pointing out the need for further studies.

## 1 INTRODUCTION

Following an increased interest in species‐specific feeding, many owners now feed their dogs with raw and more “natural” foods. Wolves are true carnivores with vegetal matter being a minor part of their diet.^1^ It is a matter of controversy whether dogs should have diets similar to that of wolves, or whether they have adapted the need for a more omnivorous diet during the thousands of years for which dogs have shared their environment with humans. Dogs share some selected genes with humans that are related to digestion and metabolism.^2^ Recent studies have shown that the amylase gene (*AMY2B*) was one of the target genes of selection during dog domestication,^3^, ^4^ and an increase in copy number variation would suggest an adaptation to a starch‐richer diet. It is likely that some dog breeds arose alongside agriculturists, and some evolved side by side with hunter‐gatherers.^5^ A study of five canine breeds (Papillon, Miniature Schnauzer, Cocker Spaniel, Labrador Retriever, and Saint Bernard) showed that, despite being of different breeds, all dogs in the study selected a 30:63:7% protein‐fat‐carbohydrate energy profile,^6^ whereas wolves preferred a 54:45:1% energy profile.^1^ One large difference between a wolf's diet and a dog's commercial dry and canned food diet is the nitrogen‐free extract (NFE) content of 0.7 g/MJ ME (megajoules of metabolizable energy) in the high protein, low carbohydrate raw diet of wolves compared with the NFE content of 25.8 and 20.0 g/MJ ME in the commercial high carbohydrate dry and canned dog foods, respectively.^1^ The NFE content represents another way to express carbohydrates and primarily originates from the starch of cereal grains.

Nutrition is now considered a fundamental issue by a growing number of medical specialties.^7^, ^8^, ^9^ This can be seen in veterinary practices, where the relationship between poor animal health and nutrition has been a growing concern. Using a raw food diet as a more natural approach for improving canine health has encountered resistance by some veterinary nutritionists,^10^, ^11^ and encouragement from others.^12^, ^13^, ^14^ Studies comparing the overall health effects, or even partial physiologic effects of heat‐processed and nonheat‐processed dry food in dogs are scarce. A recent study compared the effect of extruded, mildly cooked, and raw diets on serum biochemistry results, urinalyses, and fecal characteristics^15^ and found lower serum alkaline phosphatase activites and triglyceride concentrations and higher serum chloride concentrations  in dogs fed a raw food diet compared with those fed a dry food diet; however, all values remained within RIs. An abstract of 302 dogs fed either a raw or dry food diet found higher blood urea concentrations, hematocrits, and creatinine levels in dogs fed the raw food diet compared with those fed the dry diet, although all values remained within RIs.^16^

The objective of this pilot study was to perform a prospective comparison of two controlled commercial diets in client‐owned dogs, one being processed dry dog food and the other being nonprocessed raw dog food. We hypothesized that the different diets would have an impact on canine blood values. Only one dog breed was used to avoid genetic heterogeneity, given the vast genetic differences between breeds. Since canine atopic dermatitis (CAD) is common in Staffordshire Bull Terriers in Finland,^17^ another part of the study looked at the effects of diet on CAD.

## 2 MATERIALS AND METHODS

### 2.1 Animals and study design

This diet intervention pilot study was designed to include both atopic and healthy dogs, and most dogs in the study population had CAD. This prospective study was conducted following a classically controlled experimental design (pretest/posttest) to assess the effect of diet on blood values. All dogs were client‐owned Staffordshire Bull Terriers. They were evaluated with a clinical examination and biochemical and hematologic testing before and after the diet intervention. The clinical study was conducted in a veterinary teaching hospital at the University of Helsinki during 2013‐2014. The study protocol was approved by the Animal Experiment Board in Finland (ELLA) (permit number: ESAVI/3244/04.10.07/2013). All owners signed a written consent form. The dietary intervention lasted for 102‐188 days (median 140 days) in dogs included in these analyses.

Inclusion criteria for healthy dogs were those over 3 years of age with no skin diseases. Inclusion criteria for atopic dogs were those over 1 year of age with no other skin conditions. The diagnosis of CAD was made using currently accepted standards. Clinical status was determined using Favrot's eight criteria,^18^ and skin lesions were scored using the validated CADESI‐04 score (from 0 to 180).^19^ Owners also evaluated their dogs’ pruritus according to the validated pruritus visual analog scale (P‐VAS) (from 0 to 10).^20^ To rule out parasitic skin diseases, all dogs received parasitic treatments with three topical applications of selamectin (Stronghold; Pfizer Ltd.) every 2 weeks. Since several dogs had some skin symptoms but did not fulfill six of Favrot's eight criteria, they were classified as “borderline dogs,” and were still included in the analyses since they were present in both diet groups.

The diet intervention trial included baseline and final visits. At baseline visits, dogs underwent a thorough physical examination, and blood samples were collected. Then, the dogs were randomly divided into the two diet groups and stratified for disease severity by the sum of their CADESI‐04 and P‐VAS scores (low ≤22 or high >22), health statuses (atopic, nonatopic, and “food as a possible component”), and previous diets (≥40% of raw food, ≥80% of dry food, or neither) using a computerized randomization list.

The study diets consisted of two different commercial dog diets commonly fed in Finland. All foods have been stated as balanced and complete by the manufacturers. A heat‐processed high carbohydrate (HPHC) diet was a commercial dry dog food (Hill's Pet Nutrition, Inc.) (Table 1). A nonprocessed high‐fat (NPHF) diet was composed of either MUSH BARF Vaisto Pork‐Chicken‐Lamb or MUSH BARF Vaisto Beef‐Turkey‐Salmon or both and are commercial raw‐frozen dog foods (Mush Ltd.) (Table 2). The owners could choose either one of the NPHF diets or use both NPHF diets since many owners were concerned that their dogs were sensitive and reactive to certain animal proteins. Nevertheless, the NPHF diets were similar in macronutrient profiles as well as processing methods, making the NPHF equally different from the HPHC diet, and in this way, justifying the use of either NPHF diet as one diet (or feeding pattern). Owners were asked to feed the trial diets exclusively and to follow the amounts according to bodyweights recommended by the manufacturer. Water was allowed ad libitum. All incidental foods and supplements given during the trial period were written down in diaries given to the owners at the start of the trial. At the final visit, the same clinical protocol was followed as for the baseline visit.

**TABLE 1:** Composition and analytical constituents of Hill's Science Diet Canine Adult Sensitive Stomach & Skin with Chicken^a^ dog food (Hill's Pet Nutrition, Inc.) fed to adult Staffordshire Bull Terriers in the diet trial

Analytical constituent	In food	In dry matter
Protein (%)	25.3	27.5
Fat (%)	16	17.4
Carbohydrate (NFE) (%)	44.5	48.4
Fiber (crude) (%)	1.3	1.4
Ash (%)	4.9	5.3
Moisture (%)	8	—
Calcium (%)	0.66	0.72
Phosphorus (%)	0.58	0.63
Calcium:Phosphorus	1.1	1.1
Sodium (%)	0.35	0.38
Potassium (%)	0.64	0.7
Omega‐3 fatty acids (%)	1.2	1.3
Omega‐6 fatty acids (%)	4.8	5.2
Vitamin E (mg)	60	65
Vitamin C (mg)	7	7.6
Beta‐carotene (mg)	0.15	0.16
Additives per kg		
Vitamin A (IU)	16 000	17 391
Vitamin D (IU)	941	1023
Iron (mg)	53.7	58.4
Iodine (mg)	0.9	1.0
Copper (mg)	5.3	5.8
Manganese (mg)	5.6	6.1
Zinc (mg)	111	121
Selenium (mg)	0.15	0.16

Abbreviation: NFE, nitrogen‐free extract.

^a^ Composition (2013): Rice, maize, poultry meat meal (min. chicken 23%), maize gluten meal, dried whole egg, vegetable oil, flaxseed, digest, animal fat, potassium chloride, salt. The food is stated as complete and balanced by the manufacturer.

**TABLE 2:** Composition and analytical constituent of two nonprocessed Vaisto dog foods (Mush Ltd.) fed to adult Staffordshire Bull Terriers in the diet trial

	In food	In dry matter
Analytical constituent, pork‐chicken‐lamb^a^		
Protein (%)	15.2	38
Fat (%)	20	50
Ash (crude) (%)	4.20	10.5
Fiber (crude) (%)	0.60	1.5
Moisture (%)	60.0	0.0
Phosphorus (%)	0.65	1.6
Calcium (%)	1.09	2.7
Calcium:Phosphorus	1.7	1.7
Analyzed ingredients per kg (different batch)^c^		
Omega‐3 fatty acids (%)		0.4
Omega‐6 fatty acids (%)		3.8
Vitamin A (IU)		143 050
Vitamin D (IU)		698
Vitamin E (mg)		46.6
Iron (mg)		123
Iodine (mg)		1.86
Copper (mg)		24.2
Manganese (mg)		8.8
Zinc (mg)		119
Selenium (mg)		0.62
Analytical constituent, beef‐turkey‐salmon^b^		
Protein (%)	15.0	42.5
Fat (%)	15.8	44.8
Ash (crude) (%)	3.70	10.5
Fiber (crude) (%)	0.80	2.3
Moisture (%)	64.7	0.0
Phosphorus (%)	0.34	1.0
Calcium (%)	0.45	1.3
Calcium:Phosphorus	1.3	1.3
Analyzed ingredients per kg (different batch)^c^		
Omega‐3 fatty acids (%)		1.1
Omega‐6 fatty acids (%)		2.7
Vitamin A (IU)		80 890
Vitamin D (IU)		2130
Vitamin E (mg)		54.4
Iron (mg)		82.1
Iodine (mg)		1.64
Copper (mg)		31.5
Manganese (mg)		7.4
Zinc (mg)		79.6
Selenium (mg)		0.73

^a^ Composition (2013): (pork‐chicken‐lamb): Finnish pork 46% (meat, bone, lung, cartilage, heart, liver), Finnish chicken 29% (meat, bone, gizzard, skin, heart, cartilage, liver), Finnish lamb 20% (bone, meat, lung, cartilage, liver), vegetables 5% (spinach, broccoli, lettuce, cold‐pressed sunflower oil), egg <1%.

^b^ Composition (2013): (beef‐turkey‐salmon): Finnish beef, 47% (rumen, meat, lung, heart, cartilage, liver), Finnish turkey 38% (meat, bone, cartilage), Norwegian salmon 10% (salmon including bones), vegetables 5% (broccoli, lettuce, apple, carrot, cold‐pressed sunflower oil, camelina oil). The foods have been stated as complete and balanced by the manufacturer.

^c^ Ingredients were analysed by the manufacturer from different food batch and provided to researchers by MUSH Ltd.

### 2.2 Sample collection and laboratory analyses

Blood was collected from the jugular vein into Vacuette 3 mL EDTA and 6 mL plain serum tubes by a closed method (Vacutainer Safety‐Lok Blood collection sets; Becton Dickinson). For hematology, the blood leukocyte count (WBC), erythrocyte count (RBC), hemoglobin (Hb), mean cell volume (MCV), hematocrit (Hct) (RBC × MCV), mean cell hemoglobin (MCH) (Hb/RBC), mean corpuscular hemoglobin concentration (MCHC; Hb/Hct), and platelet count were determined from 33 EDTA blood samples. Complete blood cell counts were determined with the ADVIA 2120i Hematology System with multispecies software (Siemens Healthcare Diagnostics) and the cyanmethemoglobin method for hemoglobin measurements. Analyses were performed immediately after collection, or in very few cases (when samples were drawn late night), were stored overnight at 4°C and analyzed early in the morning.

For the biochemical analyses, the collected blood was allowed to clot and then centrifuged (2100× g, 15 minutes). The serum was used for analysis of the following analytes: ALP, ALT, albumin, bilirubin, inorganic phosphate, glucose, potassium, sodium, calcium, cholesterol, creatinine, total protein, and urea (n = 33). Measurements were performed using a Konelab 30*i* chemistry analyzer (ThermoFisher Scientific). Analyses were performed immediately or 1 day after a visit.

In addition, blood iron concentrations were determined from the lithium‐heparin whole blood samples at the MILA Laboratory (Helsinki, Finland) using inductively coupled plasma optical emission spectrometry (Iris Intrepid; Thermo Elemental) from 19 dogs. Samples were stored immediately after the visits at −20°C and analyzed 3 years later. Also, plasma folate and vitamin B_12_ were analyzed from the lithium‐heparin plasma samples from 31 dogs at the animal diagnostic laboratory Movet Oy (Kuopio, Finland) using Siemens Immulite 2000xpi. Lithium‐heparin tubes were centrifuged (2100× g, 15 minutes) immediately after the visits, and plasma was stored at −80°C. Samples were analyzed 3 years after the visits. All samples were fasting samples. Basic data and blood values are provided in the File S1.

### 2.3 Statistical analyses

To compare changes in hematologic and biochemical values during the diet intervention within the diet groups, dependent samples *t*‐tests were used if normality assumptions held, otherwise changes were tested using the Wilcoxon signed‐rank test. Depending on the normality of the data, to compare hematologic and clinical values between the two diet groups at the baseline visit, independent samples *t*‐tests or Mann‐Whitney *U*‐tests were used. Normality was assessed using the Shapiro‐Wilk test. Equality of variances was tested using Levene's test. In the case of unequal variances, Welch's *t*‐test was used. Differences between healthy dogs and dogs with atopic dermatitis were tested with similar manners. One dog in the NPHF diet group and two dogs in the HPHC diet group with corticosteroid medication were removed from the end visit ALP analysis (n = 18 and n = 12, respectively). To control for age, weight, and corticoid and NSAID medications, sex, duration of the diet, baseline blood value, and diagnosis, analysis of covariance (ANCOVA) was conducted to compare the two diet groups at the end of the study. Background characteristics were analyzed using the independent samples *t*‐test/Mann‐Whitney *U*‐test or Fisher's exact test. SPSS software (versions 22 and 25; IBM SPSS Statistics) was used for all analyses. Statistical significance was set at  *P* < .05.

## 3 RESULTS

### 3.1 Clinical characterization of the study dog population

Of the 46 dogs that finished the diet intervention study, 13 dogs were excluded. To make the study population less variable, two dogs for which the diet had changed during the trial, one that had ongoing cyclosporin medication, and five that were on the study diet less than 85 days were excluded. In addition, one each was diagnosed with localized demodicosis, hypothyroidism, and azotemia at the last visit, and were, therefore, excluded. Furthermore, two dogs were excluded from the analyses since the blood samples for hematology and clinical chemistry were available from only one of the visits. Of the remaining 33 dogs, 19 were fed the NPHF diet and 14 the HPHC diet. Eighteen dogs were suffering from CAD and had fulfilled at least six of Favrot's eight criteria. Eight dogs reported to be healthy by their owners were found to have some type of skin changes at the baseline visit, and were grouped as borderline dogs (neither were completely healthy or had clear CAD). For this reason, the number of healthy dogs in the study declined to seven dogs. The dietary intervention lasted for 102‐188 days (median 140 days) in dogs included in these analyses. There was no statistical difference between the diet groups in any of the background characteristics, including disease status and the previous diet at baseline (Table 3).

**TABLE 3:** Background characteristics of the dogs used in the analyses of hematology and serum biochemistry between the nonprocessed high‐fat diet (NPHF) and the heat‐processed high carbohydrate diet (HPHC)

Categories	NPHF diet group (N = 19)	HPHC diet group (N = 14)	*P*‐value
Dogs			
Atopic	9	9	.48
Borderline	6	2	
Healthy	4	3	
Previous diet			
≥40% raw	7	4	.58
≥80% dry	9	7	
Mixed^a^	3	3	
Age			
Years (SE)	4.8 (0.63)	5.6 (0.76)	.45
Gender			
% male	52.6	50.0	1.00
Study duration			
Days, median	140	142	.653
Weight			
Baseline, kg (SE)	17.6 (0.84)	18.2 (0.97)	.77
End visit, kg (SE)	17.5 (0.80)	18.5 (0.95)	.47
*P*‐value within groups	.63	.043*	

Note

The results are analyzed using independent samples *t*‐test/Mann‐Whitney *U*‐test, Median test or Fisher's exact test.

^a^ Meaning that it included dry and/or raw and/or home cooked food.

* *P* < .05.

#### 3.1.1 Hematology and serum biochemistry results

**FIGURE 1:**
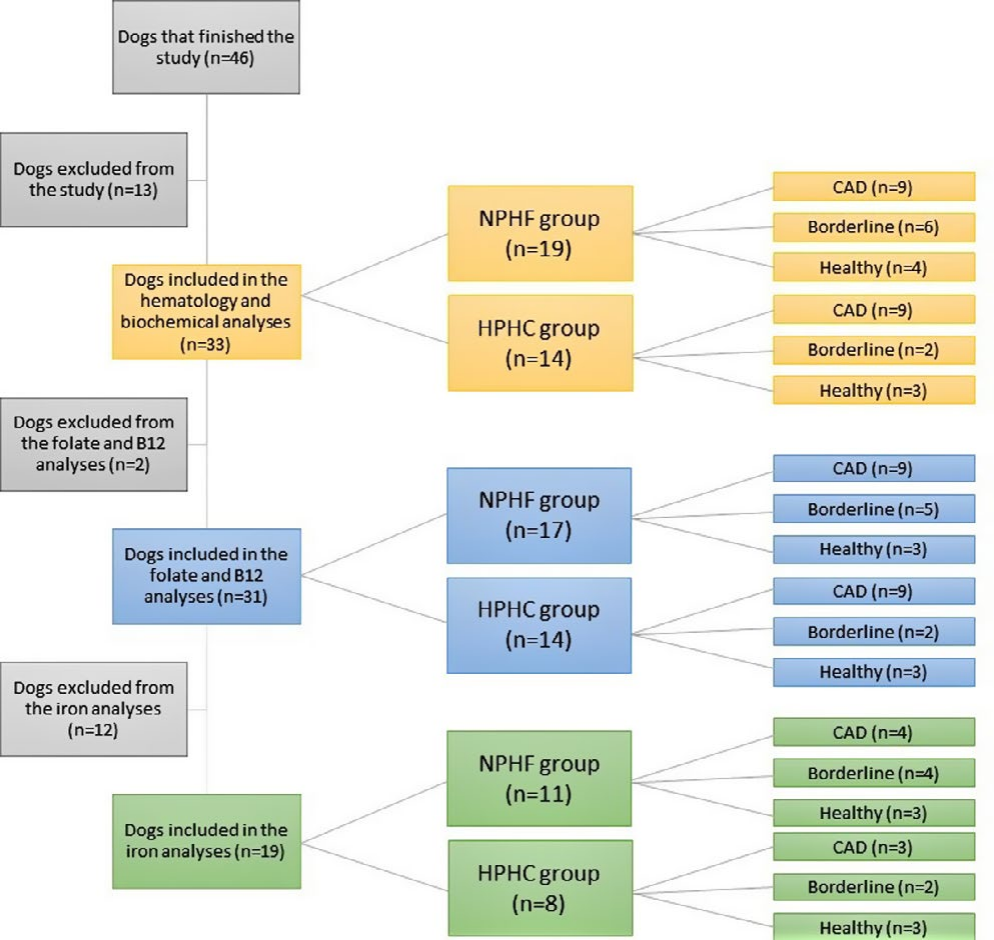
Flowchart and the distribution of the study dogs in different analyses made from the blood samples of client‐owned Staffordshire Bull Terriers. Borderline, dogs with skin symptoms that did not fulfill six of eight Favrot's criteria; CAD, canine atopic dermatitis; HPHC, heat‐processed high carbohydrate diet; NPHF, nonprocessed high‐fat diet

All the remaining 33 dogs were used in the hematology and serum biochemical analyses. The distribution of the groups can be seen in Figure 1. When atopic and healthy dogs were compared, there was no statistically significant difference in any of the serum biochemistry or hematology results before the diet intervention. After the diet intervention, only sodium values differed between atopic and healthy dogs (150.1 and 148.4 mmol/L, respectively, *P* = .03).

**FIGURE 2:**
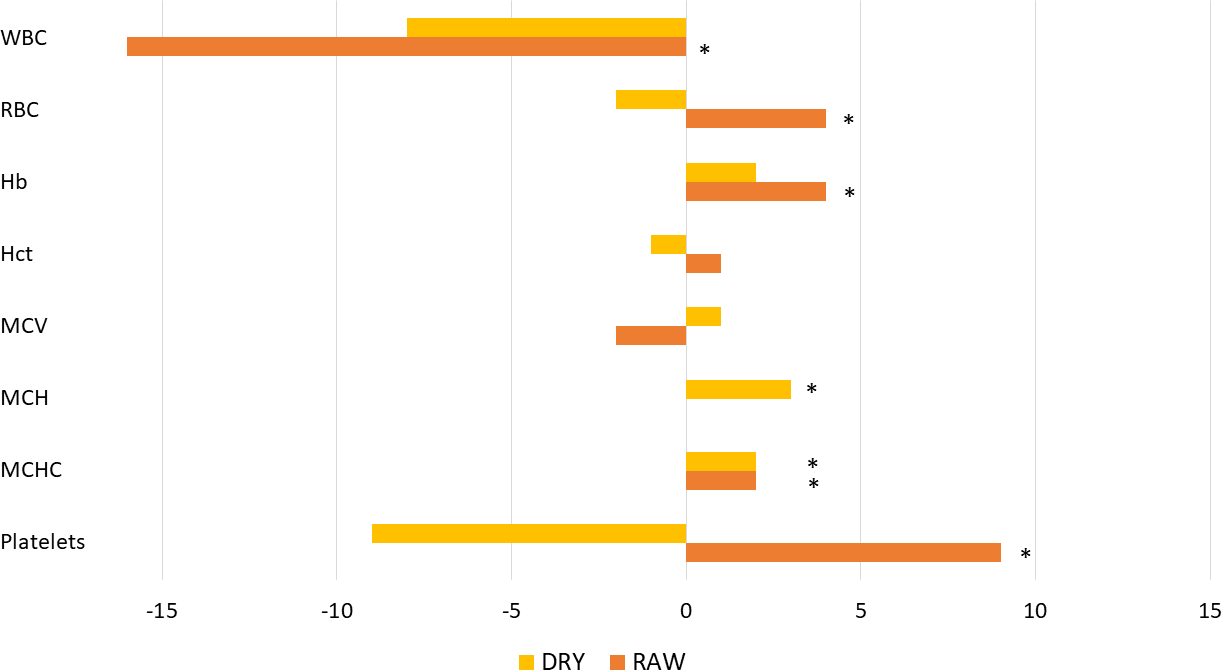
Mean change in percentage from the mean baseline values of the hematology analytes in the nonprocessed high‐fat diet (NPHF; RAW) and the heat‐processed high carbohydrate diet (HPHC; DRY) fed dogs. *Statistically significant difference. Hb, hemoglobin; Hct, hematocrit; MCH, mean cell hemoglobin; MCHC, mean cell hemoglobin concentration; MCV, mean cell volume; RBC, red blood cell count; WBC, white blood cell count

**FIGURE 3:**
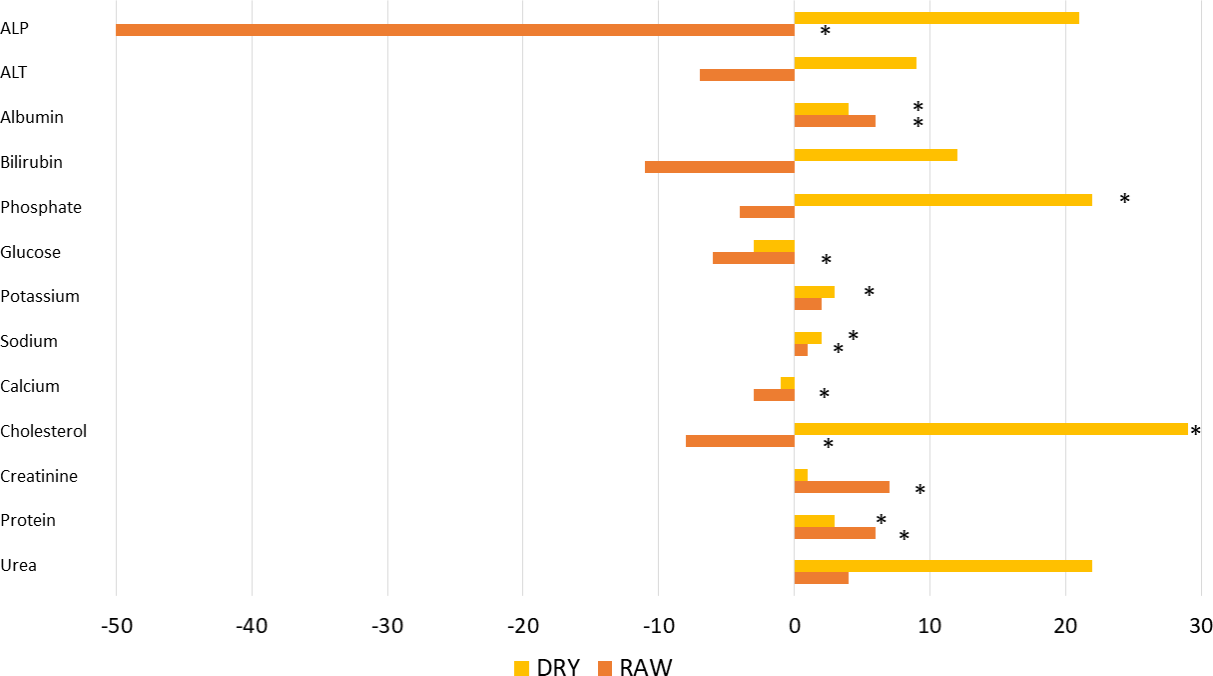
Mean change in percentage from the mean baseline values of the clinical chemistry analytes in the nonprocessed high‐fat diet (NPHF; RAW) and the heat‐processed high carbohydrate diet (HPHC; DRY) fed dogs. *Statistically significant difference. ALP, alkaline phosphatase; ALT, alanine aminotransferase

There were statistically significant changes observed in most of the hematology values within the diet groups (Table 4). In the NPHF diet group, the WBC count decreased, whereas the RBC count, Hb concentration, MCHC, and platelet count increased during the trial (Figure 2). In the HPHC diet group, the MCH and MCHC increased during the trial (Figure 3). It should be noted that the MCH was calculated using Hb and RBC values (Hb/RBC), so the statistically significant increase in MCH in the HPHC diet group was due to both a small increase in Hb and a small decrease in RBC counts. Group means for MCH and MCHC were below the RI throughout the study, and the group mean for the platelet count in the dogs fed the NPHF diet went above the RI at the final visit (Table 4).

**TABLE 4:** Hematology of the dogs in the nonprocessed high‐fat diet (NPHF) and the heat‐processed high carbohydrate diet (HPHC) groups

	RI	NPHF diet, mean ± SD (n = 19)	HPHC diet, mean ± SD (n = 14)
WBC (10^9^/L)	5.4‐17.4		
Baseline		8.82 ± 2.81	8.57 ± 2.98
End		7.45 ± 1.78	7.85 ± 2.48
*P*‐value		.008*	.31
RBC (10^12^/L)	5.3‐8.0		
Baseline		7.34 ± 0.66	7.09 ± 0.42
End		7.64 ± 0.71	6.98 ± 0.60
*P*‐value		.043*	.54
Hb (g/L)	140‐203		
Baseline		169 ± 16.7	162 ± 11.1
End		176 ± 14.1	165 ± 11.4
*P*‐value		.034*	.92
Hct (%)	38‐57		
Baseline		51.3 ± 5.11	50.0 ± 3.62
End		52.0 ± 4.10	49.7 ± 3.70
*P*‐value		.48	.43
MCV (fL)	67‐80		
Baseline		69.9 ± 3.89	70.5 ± 3.02
End		68.3 ± 4.10	71.4 ± 2.72
*P*‐value		.13	.16
MCH (pg)	24‐29		
Baseline		23.1 ± 1.08^a^	22.9 ± 0.89^a^
End		23.2 ± 1.25^a^	23.7 ± 0.92^a^
*P*‐value		.77	.001*
MCHC (g/L)	345‐367		
Baseline		331 ± 10.6^a^	325 ± 12.1^a^
End		339 ± 7.44^a^	333 ± 8.50^a^
*P*‐value		.015*	.012*
Platelets (10^9^/L)	102‐395		
Baseline		374 ± 81.7	362 ± 110
End		409 ± 63.8^b^	331 ± 112
*P*‐value		.012*	.40

Note

The results are analyzed using dependent samples *t*‐test/Wilcoxon signed‐rank test.

Abbreviations: Hb, hemoglobin; Hct, hematocrit; MCH, mean cell hemoglobin; MCHC, mean cell hemoglobin concentration; MCV, mean cell volume; RBC, erythrocytes; WBC, leukocytes.

^a^ Group mean is below the RI.

^b^ Group mean is above the RI.

* *P* < .05.

Significant changes were also observed in most of the biochemical values inside the diet groups (Table 5). Within the NPHF diet group ALP, glucose, total calcium, and cholesterol decreased significantly, whereas albumin, potassium, sodium, creatinine, and total protein increased significantly (Figure 2). Within the HPHC diet group, albumin, inorganic phosphate, sodium, cholesterol, and total protein increased significantly (Figure 3).

**TABLE 5:** Clinical chemistry of the dogs in the nonprocessed high‐fat diet (NPHF) and the heat‐processed high carbohydrate diet (HPHC) groups

	RI	NPHF diet, mean ± SD (n = 19)	HPHC diet, mean ± SD (n = 14)
ALP (U/I)	33‐215		
Baseline		99.3 ± 49.3	80.3 ± 44.6
End		49.4 ± 19.4^a^	102.2 ± 41.7^b^
*P*‐value		<.001*	.075
ALT (U/I)	18‐77		
Baseline		53.8 ± 27.5	55.8 ± 15.7
End		50.0 ± 24.8	60.7 ± 23.5
*P*‐value		.26	.59
Albumin (g/L)	30‐41		
Baseline		31.6 ± 1.61	32.1 ± 2.14
End		33.6 ± 2.11	33.5 ± 2.00
*P*‐value		<.001*	.025*
Bilirubin (μmol/L)	2.5‐8.5		
Baseline		3.33 ± 1.37	2.89 ± 0.82
End		2.98 ± 0.84	3.23 ± 0.87
*P*‐value		.36	.27
P (mmol/L)	0.93‐2.25		
Baseline		1.01 ± 0.23	0.88 ± 0.21^c^
End		0.97 ± 0.25	1.07 ± 0.23
*P*‐value		.53	.009*
Glucose (mmol/L)	4.0‐6.4		
Baseline		5.57 ± 0.48	5.44 ± 0.50
End		5.26 ± 0.39	5.28 ± 0.35
*P*‐value		.044*	.13
K (mmol/L)	4.2‐5.4		
Baseline		4.33 ± 0.23	4.23 ± 0.39
End		4.43 ± 0.22	4.34 ± 0.36
*P*‐value		.017*	.14
Na (mmol/L)	147‐157		
Baseline		148 ± 1.90	147 ± 2.19
End		150 ± 1.45	150 ± 1.23
*P*‐value		.006*	.006*
Tot Ca (mmol/L)	2.3‐3.0		
Baseline		2.70 ± 0.05	2.70 ± 0.09
End		2.63 ± 0.11	2.66 ± 0.12
*P*‐value		.013*	.16
Chol (mmol/L)	3.7‐9.8		
Baseline		6.88 ± 1.65	6.51 ± 1.46
End		6.33 ± 1.11	8.37 ± 1.86
*P*‐value		.039*	.008*
Crea (μmol/L)	57‐116		
Baseline		87.7 ± 9.03	83.4 ± 9.58
End		94.1 ± 8.29	84.4 ± 10.4
*P*‐value		.001*	.72
Tot protein (g/L)	58‐77		
Baseline		57.4 ± 2.81^c^	58.6 ± 4.36
End		60.8 ± 7.28	60.7 ± 2.30
*P*‐value		.026*	.038*
Urea (mmol/L)	2.4‐8.8		
Baseline		5.48 ± 2.04	4.59 ± 0.78
End		5.71 ± 1.24	5.61 ± 1.54
*P*‐value		.64	.093

Note

The results are analyzed using dependent samples *t*‐test/ Wilcoxon signed‐rank test.

Abbreviations: ALP, alkaline phosphatase; ALT, alanine aminotransferase; Chol, cholesterol; Crea, creatinine; K, potassium; Na, sodium; Tot Ca, total calcium.

^a^ One dog with corticosteroid medication removed (n = 18).

^b^ Two dogs with corticosteroid medication removed, (n = 12).

^c^ Group mean is below the RI.

* *P* < .05.

At the baseline visit, there were no statistically significant differences between the two diet groups. Erythrocyte counts, MCHC, and thrombocyte counts were found to be greater in dogs fed the NPHF diet compared with those that were fed the HPHC diet after the diet intervention (Table 6). Conversely, MCV, MCH, ALP, phosphate, and cholesterol were found to be greater in dogs fed the HPHC diet compared with those fed the NPHF diet (Table 6).

**TABLE 6:** Difference in hematology and biochemistry, folate, B_12_ and iron between the nonprocessed high‐fat diet (NPHF) and the heat‐processed high carbohydrate diet (HPHC) fed dogs

	NPHF diet, adjusted mean ± SD (n = 19)	HPHC diet, adjusted mean ± SD (n = 14)	*P*‐value
WBC (10^9^/L)	7.63 ± 0.85	8.45 ± 0.75	.29
RBC (10^12^/L)	7.52 ± 0.29	6.96 ± 0.25	.04*
Hb (g/L)	173.0 ± 5.8	163.5 ± 4.9	.08
Hkr (%)	50.6 ± 1.8	48.5 ± 1.5	.21
MCV (fL)	67.2 ± 1.5	70.5 ± 1.3	.02*
MCH (pg)	23.0 ± 0.4	23.7 ± 0.3	.03*
MCHC (g/L)	344.8 ± 4.0	336.5 ± 3.4	.03*
Platelets (10^9^/L)	386.1 ± 38.3	311.0 ± 33.6	.04*
ALP (U/I)^a^	46.7 ± 8.9	109.0 ± 9.6	<.001*
ALT (U/I)	47.4 ± 7.9	53.3 ± 6.6	.40
Alb (g/L)	33.2 ± 0.8	32.7 ± 0.7	.44
Bilirubin (μmol/L)	2.80 ± 0.37	3.04 ± 0.32	.47
P (mmol/L)	0.97 ± 0.10	1.19 ± 0.10	.02*
Glucose (mmol/L)	5.44 ± 0.16	5.44 ± 0.14	.98
K (mmol/L)	4.43 ± 0.08	4.41 ± 0.08	.75
Na (mmol/L)	149.3 ± 0.6	149.1 ± 0.5	.68
Tot Ca (mmol/L)	2.63 ± 0.04	2.61 ± 0.03	.65
Chol (mmol/L)	5.88 ± 0.55	8.42 ± 0.50	<.001*
Creat (μmol/L)^d^			
Tot protein (g/L)	65.5 ± 2.4	63.2 ± 2.1	.29
Urea (mmol/L)	6.79 ± 0.53	6.36 ± 0.47	.38
Folate (ng/mL)^b^	5.0 ± 1.5	9.6 ± 1.2	.001*
B_12_ (pg/mL)^b^	363 ± 91	562 ± 72	.01*
Iron (mmol/L)^c^	9.9 ± 0.5	9.3 ± 0.6	.26

Note

Results were analyzed using ANCOVA controlled for age, weight, corticoid and NSAID medication, gender, duration time of the diet, baseline blood value and diagnosis.

Abbreviations: ALP, alkaline phosphatase; ALT, alanine aminotransferase; Chol, cholesterol; Crea, creatinine.

^a^ Three dogs with corticosteroid medication removed (raw diet, n = 18; dry diet, n = 12).

^b^ 31 dogs used for the analyses (raw diet, n = 17; dry diet, n = 14)

^c^ 19 dogs used for the analyses (raw diet, n = 11; dry diet, n = 8).

^d^ Not normally distributed.

* *P* < .05.

#### 3.1.2 Folate, B_12_, and iron

Plasma folate and B_12_ concentrations were measured in 31 dogs since there was no plasma left from two remaining dogs for the analyses. The group distributions can be seen in Figure 1. The mean plasma folate concentration (RI 3‐15 ng/mL) in the NPHF diet group at baseline and during the final visit was 12.0 ng/mL (SD ± 5.9) and 5.0 ng/mL (SD ± 3.8), respectively (*P* = .001). In the HPHC diet group, the baseline and final visit concentrations were 10.8 ng/mL (SD ± 5.9) and 8.4 ng/mL (SD ± 1.8), respectively (*P* = .13). During the final visit, in the NPHF group, folate concentrations fell below the RI in four dogs and surpassed the RI in one dog. The plasma folate concentrations were significantly higher in dogs fed the HPHC diet compared with those fed the NPHF after the diet intervention (Table 6).

The mean plasma B_12_ concentration (RI 200‐800 pg/mL) in the NPHF diet group at baseline and during the final visit was 620 pg/mL (SD ± 275) and 457 pg/mL (SD ± 178), respectively (*P* = .01). In the HPHC diet group, the baseline and final visit concentrations were 574 pg/mL (SD ± 249) and 584 ng/mL (SD ± 200), respectively (*P* = .89). None of the dogs had B_12_ concentrations that fell below the RI; however, one dog (the same dog that surpassed the folate RI) in the NPHF diet group and three dogs in the HPHC diet group had B_12_ concentrations that surpassed the RI at the final visit. The plasma B_12_ concentration was significantly higher in dogs fed the HPHC diet compared with those fed the NPHF diet after the diet intervention (Table 6).

Whole blood iron was measured from 19 dogs that had enough blood left over for this analysis. The distribution of the dogs into groups is shown in Figure 1. The mean whole blood iron concentration in the NPHF diet group was significantly lower at the final visit (9.7 mmol/L, SD ± 0.9) compared with the baseline visit (10.6 mmol/L, SD ± 1.0; *P* = .026) after the diet intervention. In the HPHC diet group, no significant changes were observed (*P* = .12) between the baseline visit (9.7 mmol/L, SD ± 0.8) and the final visit (9.0 mmol/L, SD ± 0.5). The ANCOVA analysis between the diet groups after the diet intervention showed no significant differences (Table 6).

## 4 DISCUSSION

This study shows that different diets induced different types of changes in the hematologic and biochemical variables of dogs, as well as in blood iron, B_12_, and folate measurements. However, most of the changes seen in this study occurred within the RIs. The findings of higher erythrocyte counts and hemoglobin levels in the dogs of the NPHF group were comparable to the results of a study by Kronfeld et al,^21^ who verified higher RBC counts and Hb levels in dogs fed the highest protein diets compared with lowest protein diets; in this study, the NPHF diet had a higher protein concentration than the HPHC diet. Algya et al^15^ found no differences in the hematologic profiles between dogs fed a dry vs raw food diet, but the protein contents of those diets did not differ (24.07% dry matter [DM] in the dry diet and 25.13% DM in the raw food). Active erythropoiesis requires adequate amounts of protein^22^ as well as iron, folate, and vitamin B_12_.^23^ Since the NPHF diet provided more protein, which is known to increase erythropoietin production, the primary regulator of erythropoiesis,^22^ it might have contributed to the increased erythropoiesis in dogs fed the NPHF diet. Increased erythrocyte production uses the body's iron stores^24^, ^25^ since about 75% of the iron present in plasma is transported to the bone marrow to assist in the development of new erythroid cells.^26^ Erythrocytes accumulate folate only during erythropoiesis,^27^ which could lead to decreased serum concentrations. There were no significant changes seen in plasma folate and vitamin B_12_ concentrations in the dogs fed the HPHC diet, suggesting that there was neither increased folate and B_12_ demand nor a dietary deficiency present in the HPHC diet group. In fact, three dogs surpassed the B_12_ RI. When samples are stored for a long time, B_12_ concentrations can decrease,^28^ and this makes the comparison of these results to RIs uncertain. In contrast, the blood concentration of these vitamins decreased significantly in the dogs of the NPHF diet group during the trial, and four dogs fell below folate RIs. Folate concentrations could be considered stable even after long storage times at −80°C.^28^ Decreases in these vitamin concentrations could indicate an increased demand or dietary deficiency. The latter is highly unlikely since the manufacturer states that, in the NPHF diets, folate contents were 417 µg/100 g and 278 µg/100 g DM, and B_12_ contents were 22.9 µg/100 g and 11.8 µg/100 g DM, which fulfill the recommended allowances for the adult dog.^29^ Whole blood iron concentrations decreased in both diet groups, although this decrease was only significant in the NPHF diet group, even though the absorption of heme‐iron, present in meat, is five to ten times greater than that of nonheme iron, present in grains and vegetables.^30^ The average lifespan of canine erythrocytes is 86‐106 days,^31^ and as the median study duration time was 140 days, it should have been enough for iron deficiency to be seen. In puppies, 30 days is considered enough time to estimate the minimum iron requirements,^32^ which might be an underestimate for adult dogs. Nevertheless, in veterinary medicine, dietary iron deficiency in adult dogs is rarely seen.^33^ Iron content of the NPHF diets was higher than that added to the HPHC diet as the additive, E1; however, the exact additive compound was not mentioned. Some inorganic sources of iron have very poor bioavailability,^29^ which could have affected the decreased blood concentrations in the HPHC diet group. On the other hand, in the NPHF group, the decrease in blood iron might have been because more iron was needed for the production of erythrocytes^25^ since the heme iron present in the NPHF diets should have fulfilled the recommended allowance for the dogs.^29^ Reference intervals of the whole blood iron for dogs have been reported only in Golden Retrievers,^34^ ranging from 9.87 mmol/L to 11.1 mmol/L. In this study, the mean value for iron in both diet groups fell below 9.87 mmol/L, but since the diets of the Golden Retrievers were not reported and some breed differences could exist, these results might not be comparable. Taken together, the increase in RBC count and Hb in the NPHF diet‐fed dogs indicate increased erythropoiesis, which could have influenced to decreased levels of iron, folate, and B_12_ concentrations in these dogs. In addition to this, it should be noticed that intestinal bacteria can produce folate and use B_12_.^35^ Different diets have been recently reported that can modify gut microbiota in dogs,^36^, ^37^ and thus, the decreased plasma B_12_ concentrations in the NPHF diet‐fed dogs might also be due to alterations in gut microbiota, which should be studied further.

Although the calculated MCH and MCHC values were below RI in almost all dogs in this study, they seem to be consistent with the results of Staffordshire Bull Terriers in a study of Lawrence et al,^38^ who reported hematologic values for several different breeds. In that study, the mean MCH and mean MCHC was 23.35 pg and 33.35 g/dL, respectively, in Staffordshire Bull Terriers that were both within the RI used in that study. In fact, breed‐specific RIs for MCHC have been proposed for Greyhounds^39^ and Bernese Mountain dogs,^40^ so the MCH and MCHC reported in our study might be a breed‐specific feature for Staffordshire Bull Terriers. However, it should be noted that different types of cell counters used in different studies could also have an impact on the results. In addition, this study was not conducted using only healthy dogs, which could affect the results, so this should be studied further with larger numbers of healthy dogs.

The activity of ALP decreased in the NPHF diet group and was significantly higher in the HPHC diet than NPHF diet‐fed dogs at the final visit. Increased ALP in adult dogs is usually a sign of functionally stimulated tissues, particularly the liver,^41^ and when markedly elevated, it is associated with a variety of diseases.^42^ In this study, however, the change in either of the diet groups was not in an order of magnitude that could be linked to these conditions. Algya et al^15^ also reported significantly higher ALP in dogs fed a dry diet than in dogs fed a raw diet. In addition, a high‐fat diet has been reported to lower ALP more than a low‐fat or low‐protein diet in dogs,^43^ and in this study, the NPHF diets included more fat (44.8%‐50% in DM) than the HPHC diet (17.4% in DM). A protein‐deficient diet has been reported to increase serum ALP in dogs,^44^ but in this study, the HPHC diet included an appropriate amount of protein (27.5% in DM) according to the Association of American Feed Control Officials (18% in DM).^45^ Swanson et al^46^ reported that in dogs that were fed a meat‐based diet, ALP increased over time, but in old dogs that were fed a plant‐based diet, ALP remained the same. In that study, however, both diets consisted of processed dry food, unlike in our study, which might have influenced the results. ALP is responsible for liberating inorganic phosphate,^47^ and serum inorganic phosphate levels increased in the HPHC diet group alongside with ALP activity. Serum inorganic phosphate levels in the dogs fed the HPHC diet was also significantly higher than in the dogs fed the NPHF diet, although the phosphorus content in the NPHF diet was higher than in the HPHC diet (1.0/1.6 in DM vs 0.63 in DM, respectively). This indicates that increased ALP activity would more likely be the explanatory factor behind the inorganic phosphate levels. A positive correlation between ALP and C‐reactive protein, a marker of inflammation, has been reported in humans,^48^ and both increased ALP and phosphate, even within the RI, has been associated with increased total mortality in humans.^49^ The same kind of epidemiologic data does not exist in dogs, which calls for further research in this area. The reason for the opposite changes in ALP activity and serum inorganic phosphate levels in the two diet groups of this study remains unclear and should be studied further. Measurements of ALP isoenzymes might provide some explanation for ALP results, but those analyses could not be performed in this pilot study.

Serum cholesterol levels decreased in the NPHF diet group and increased in the HPHC diet group during the dietary intervention. Three dogs in the HPHC diet group even surpassed the upper RIs for cholesterol, even though the NPHF foods included more meat and animal‐based fats. In a study conducted by Hansen et al,^50^ dogs suffering from chronic renal failure fed a high‐protein dry food had higher blood cholesterol values than dogs fed a low‐protein dry food. Swanson et al^46^ reported elevated blood cholesterol levels in a group of dogs fed an animal product‐based diet compared with a group of dogs fed a plant product‐based diet. Kronfeld et al^21^ reported increased blood cholesterol in dogs fed a diet with high protein and fat content, and Algya et al^15^ found no difference in cholesterol concentrations between dogs fed a raw food diet and those fed a dry food diet. Our results contradicted all of these canine study results. In the first three studies, dry dog food diets with differing protein or fat content were used; thus, their results are not directly comparable with ours. More research is needed regarding the effects of the diet on the blood cholesterol of dogs, as the results from different studies are still controversial.

Between atopic and healthy dogs, only sodium values differed after the diet intervention. Since this difference was not present at the baseline visit, it is unlikely related to the disease itself and should be considered as a coincidental finding.

There are limitations to this study. The number of dogs was small, but the results of this pilot study showed that there is a need for future research on the effect of feeding practices and dietary ingredients on canine health. The fact that the study included 18 atopic dogs, eight borderline dogs, and only seven healthy dogs makes the results hard to interpret. For this reason, there is a possibility that the underlying atopy has an impact on the results, although there were no differences between atopic and healthy dogs in any other values than sodium. Also, the previous diet fed before the intervention varied between the study dogs. However, to avoid the effect of the disease or previous diet on the results, the dogs were equally stratified into the two diet groups. A recent study by Algya et al,^15^ using healthy dogs, supports our findings related to higher ALP activity in the HPHC food fed dogs. It is difficult to say what aspect of the diets might have caused the changes seen in this study, as there were many differences in the ingredients, minerals, and vitamins of the food, as well as in the processing of the food. The NPHF diet had a much higher protein and fat content, whereas the HPHC food diet had a higher carbohydrate content. The animal protein sources also differed between the diets. However, the aim of this study was to find out if two very different feeding practices would have an impact on blood values, and in fact, many differences were seen. Nevertheless, these results only apply to the diets used in this study, and the effects should be further studied using different types of raw and dry diets, and diets differing in fat and carbohydrate content. Also, the study dogs were client‐owned and not living in a controlled environment, which made it impossible to control for all other possible foods that were given to the dogs, although owners were asked to report all other food given and to feed the dogs the study food exclusively. However, results of client‐owned dogs might reflect more closely the reality of the target population to which pet foods are intended. The length of this study (median 140 days) should have been adequate to see changes in the blood values, since Algya et al^15^ saw differences by 18 days after the start of the study, and Hansen et al^50^ saw changes within 56 days of the study start. The study by Kronfeld et al^21^ lasted 196 days, and the study by Swanson et al^46^ was conducted over 1 year with sample collections at 3, 6, 9, and 12 months. Differences in many values could already be seen after 3 or 6 months.

In conclusion, this study presents information about the effects of two different diets on changes in hematology and biochemistry analytes, as well as changes in blood folate, B_12_, and iron levels in dogs. The diet type was shown to have a significant effect on many of the blood analytes, although most analytes remained within the RIs. The results from this study will be of value for future studies looking at the impact of different feeding practices on canine physiology.

## Supporting information

Supplementary Material

Supplementary Material
