# Supplementary material for: The effect of a raw vs dry diet on serum biochemical, hematologic, blood iron, B12, and folate levels in Staffordshire Bull Terriers
Source: Vet Clin Pathol. 2020 Apr 23;49(2):258–69. doi: 10.1111/vcp.12852 (PMC7383997; doi:10.1111/vcp.12852)
Supplement: Supplementary file 2 — Supplementary Material [file VCP-49-258-s002.docx]

**Supporting information**

**S1.** Basic data and blood values of each study dog.
